# A Case of Osteodiscitis in a Preschool-Aged Child

**DOI:** 10.7759/cureus.12835

**Published:** 2021-01-21

**Authors:** Deepa Vasireddy, Jibran E Atwi

**Affiliations:** 1 Pediatrics, Pediatric Group of Acadiana, Lafayette, USA

**Keywords:** discitis, osteodiscitis, back pain, pediatric, child

## Abstract

Infectious discitis and osteodiscitis in children are rare. The usual age of occurrence is between two and five years. The diagnosis is most often delayed due to mild presenting symptoms.

We present the case of a five and half year old child who presented with progressively worsening lower back pain over a period of two weeks, which was made worse with bending forward. He did not have fever, pain in his lower extremities, or any other accompanying symptoms. He did not have a history of recent illnesses or trauma to the lower back. The laboratory work revealed an elevated erythrocyte sedimentation rate (ESR) and C-reactive protein (CRP), and rest of the parameters were within normal limits. CT scan of his spine showed findings of well-defined defects on the endplates of L4-L5 with prevertebral soft tissue thickening. MRI with and without contrast of the spine confirmed the findings and detected mild focal erosive changes at the opposing endplates of L4-L5 with disc space narrowing, thin fluid along the anterior margin of the disc, and shallow disc bulging, which were consistent with osteodiscitis.

A conservative approach with intravenous antibiotics followed by a switch to oral antibiotics was undertaken with good clinical recovery.

Treatment strategy for osteodiscitis in children is generally antibiotic therapy. Prognosis in children is good. In some cases based on the pain severity, other measures such as bed rest, analgesics, and casting for immobilization may be required. Biopsy tends to be reserved in uncertain cases or in those that have poor response to pain control measures and antibiotics.

## Introduction

Discitis and osteodiscitis are relatively rare in children. The incidence rate is yet to be established. It does not appear to have a sex predilection based on current pediatric literature. The most frequent cause is due to pathogenic microorganisms. It usually occurs in children between two and five years of age [1]. Most children tend to have symptoms for a prolonged period before the diagnosis is made.

The hematogenous spread of these microorganisms from a distant focus through the blood vessels supplying the vertebral endplates and the annulus fibrosus of the vertebral disc allows them to infect these structures [2,3]. The common organisms that tend to cause acute osteodiscitis are *Staphylococcus aureus*, *Staphylococcus epidermidis*, *Kingella kingae*, *Enterobacteriaceae*, and *Streptococcus pneumoniae* [4]. Staphylococcus aureus is the most common pathogen. Subacute and chronic cases can be caused by organisms such as *Mycobacterium tuberculosis*, *Brucella species*, *Aspergillus species*, *Candida* species, and *Cryptococcus neoformans* [5]. The yield of isolating the causative organisms from the blood cultures, tissue aspiration, and biopsy specimens is low.

We present the case of a child who presented with a solitary symptom of lower back pain. The patient’s identification has been kept confidential in the article.

## Case presentation

A five years and six months old African American male child presented to his pediatrician in the outpatient clinic with the solitary complaint of lower back pain. The back pain was located in the midline, in the lower lumbar region. Pain had set in two weeks prior to his clinic visit. Initially his pain was intermittent.

It seemed to be exacerbated when the child bended forward. The mother started noticing the child would sit with his back stiff in the chair. He would also walk slowly holding his back straight. He seemed hesitant to bend down to pick up his toys and would ask for assistance. Into the second week of illness, his back pain was more constant throughout the day. Acetaminophen and ibuprofen would relieve the pain temporarily with recurrence after. Pain was non-radiating, and intensity was described as 7/10 by the child. He did not have paresthesias.

At night, he started having discomfort maneuvering in bed while sleeping and would frequently wake up in pain. The child was afebrile through his course of illness. No trauma to that area of the body could be recalled by the child and parents. There were no overlying skin changes noted at home. He did not have a limp. The child did not complain of any extremity or other joint pain. He did not have any abdominal pain, history of constipation, or rash. He denied dysuria, and no hematuria was noted at home. He did not have any other accompanying symptoms. There was no history of recent travel. The parents could not recollect any known sick contacts. There was no known family history of rheumatological or autoimmune diseases.

He was evaluated by the pediatrician in the clinic, with physical examination being notable for decreased forward flexion and lateral rotation of back and tenderness on palpation of his lower lumbar region in the midline. He stayed afebrile. Complete blood count was within normal limits. Erythrocyte sedimentation rate (ESR) was noted to be elevated at 66 mm/hr. The child was directed to the emergency room for further evaluation. In the emergency department, physical examination was consistent with that of the pediatrician. C-reactive protein (CRP) was found to be elevated at 25.3 mg/mL. Urinalysis was unremarkable. Comprehensive metabolic profile, which includes liver and renal function tests, was within normal limits as well. Creatinine kinase was non-elevated. Blood and urine cultures were obtained.

**Figure 1:**
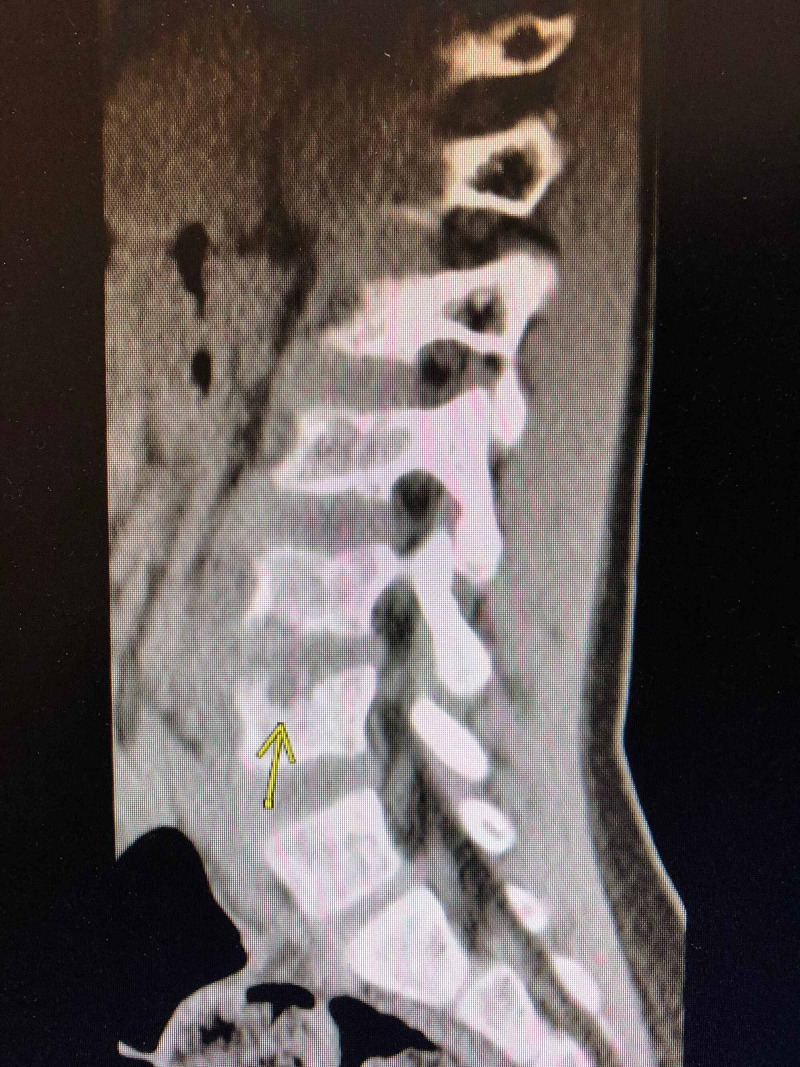
CT scan showing erosive changes in L5.

**Figure 2:**
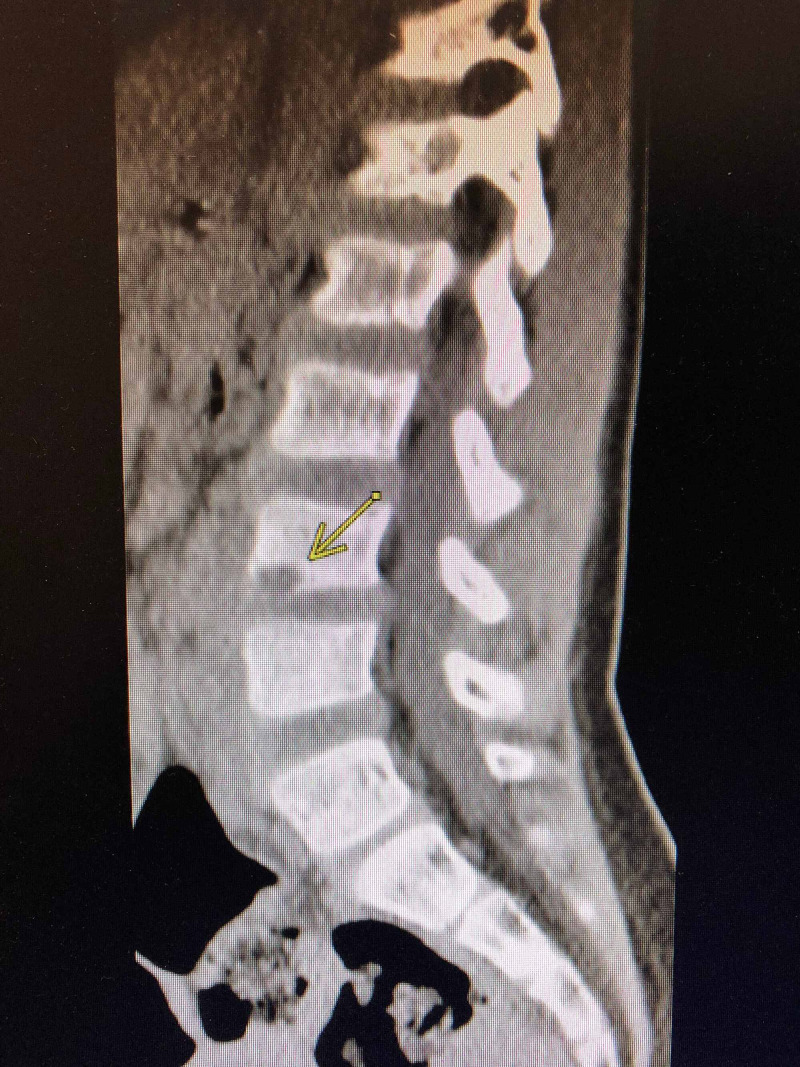
CT scan showing erosive changes in L4.

**Figure 3:**
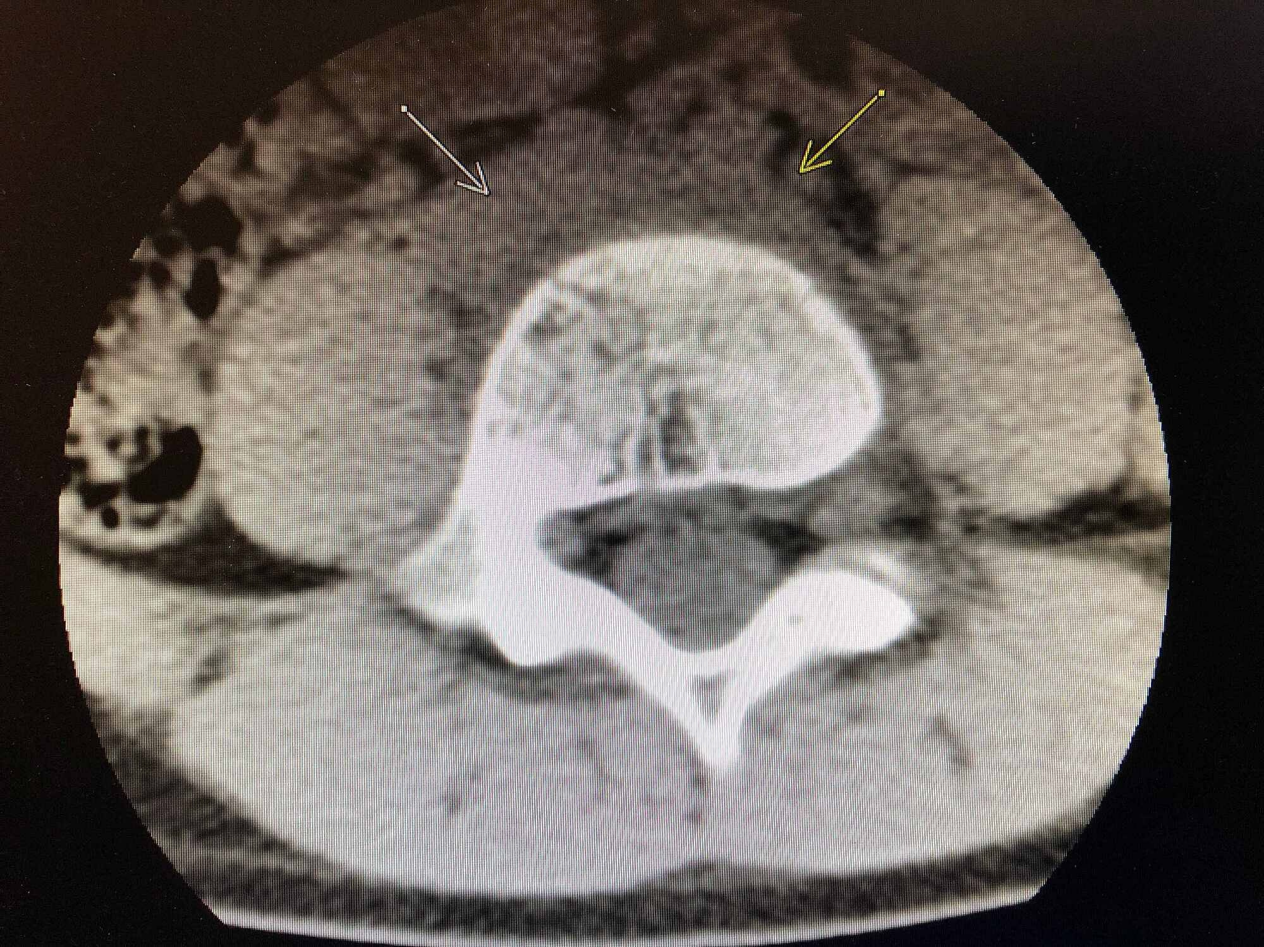
CT scan showing abnormal soft tissue thickening and edema anteriorly centered at the L4-L5 disc space level.

Computed tomography (CT) scan of his spine was performed, which showed prevertebral soft tissue thickening at L4-L5 with two well-formed defects involving the inferior and superior endplates of L4 and L5 (Figures 1-3).

**Figure 4:**
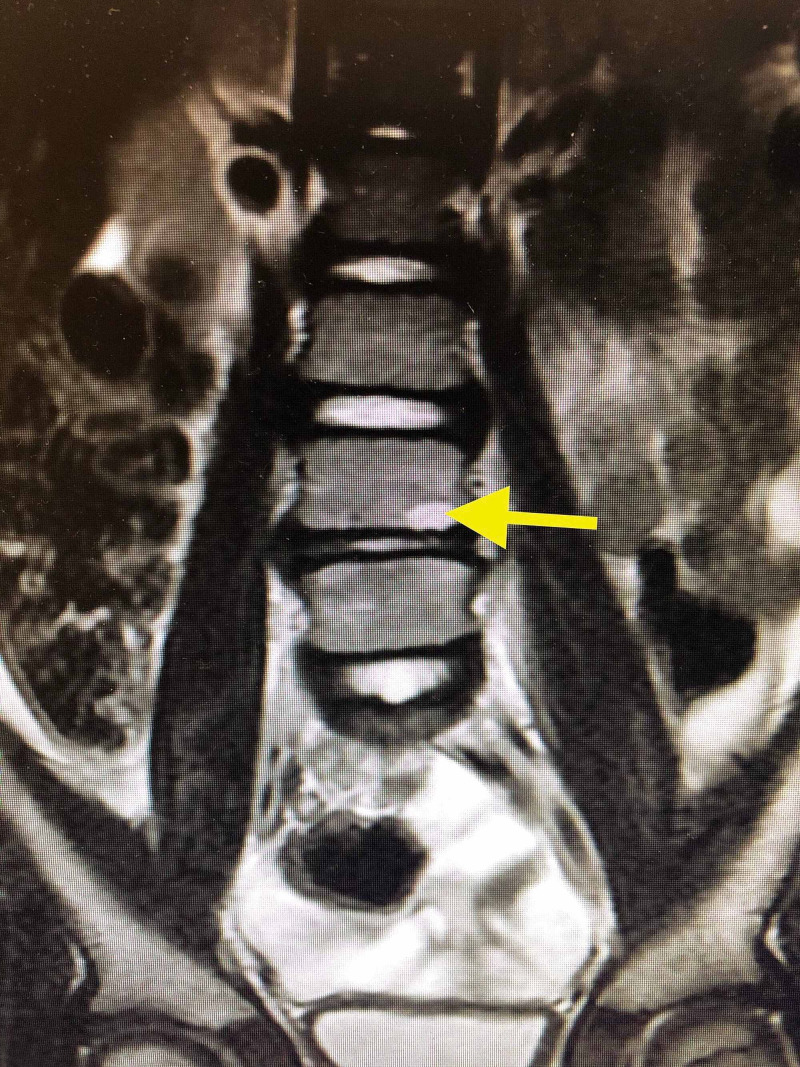
MRI scan showing vertebral erosive changes.

Magnetic resonance imaging (MRI) with and without contrast was recommended by the radiologist, which was conducted after keeping the child in nil per os (NPO) status overnight for sedation needed for the MRI. MRI findings were reported as mild disc space narrowing at the L4-L5 level with thin fluid along the anterior margin of the disc, abnormal soft tissue thickening and edema anteriorly centered at the L4-L5 disc space level, and shallow disc bulging, which were consistent with osteodiscitis (Figure 4).

An orthopedic was consulted, who agreed with conservative management with antibiotics. Intravenous clindamycin and ceftriaxone was initiated as the initial antibiotic regimen after drawing the blood culture. Antibiotic selection was based on local sensitivity patterns, and clindamycin achieves good drug concentrates in the bone. Purified protein derivative was placed during his admission, which was read as negative at 72 hours. Blood culture and urine culture did not show any growth. Per infectious disease recommendations, antibiotic was switched to a monotherapy of intravenous clindamycin, and after completion of five total days of intravenous therapy, he was switched to per oral clindamycin for three more weeks. Into the day of discharge from the hospital, the ESR had declined to 58 mm/hr and CRP had declined to 18.4 mg/mL. Pain during his course of admission was being well controlled with acetaminophen as needed, and after being discharged home he did not require medications for pain control. The child showed complete resolution of symptoms by three weeks since the onset of illness. ESR was rechecked after completion of four weeks of antibiotics, and due to continued elevation, it was checked at two-week intervals till the ESR was less than 20 mm/hr, which was the goal for discontinuation of antibiotic therapy. A repeat MRI was obtained 10 weeks from the initial MRI, which showed residual inflammation in L4-L5 with mild paraspinal enhancement. No drainable fluid collection was seen. The radiologist suggested a follow-up scan in two to three months to reevaluate for resolution.

## Discussion

The lumbar region of the spine is one of the commonest affected areas by discitis and osteodiscitis [6]. The lumbar and lumbosacral areas have the highest incidence of involvement in the spine. It has been noted that in children younger than 5 years, the lumbar spine is more commonly involved [7]. A gender predilection has not been ascertained in the pediatric literature. The symptoms of discitis can be subtle, thus delaying the diagnosis [8,9]. Fever may not be a presenting symptom. Back pain and hesitancy in walking are the common symptoms. Lower extremity pain or weakness and abdominal pain are less common accompanying symptoms.

There are several differential diagnoses for discitis, such as disc conditions such as disc degeneration and herniation, Scheuermann's kyphosis, spondylolysis, spondylolisthesis, traumatic injuries, muscular spasm, tumors, and sickle cell crisis or vaso-occlusive crisis [10]. Laboratory findings are most often within normal limits, except for varying degrees of elevation of inflammatory markers such as ESR and CRP [11,12]. MRI is the preferred modality in suspected cases of discitis [13,7]. Plain radiograph tends to not show any changes in early stages of the disease [14]. CT scan is useful in guiding biopsies in cases that warrant it [15]. Antibiotic therapy is the mainstay of treatment in most cases without complications [16]. Anti-Staphylococcal antibiotic and a third-generation cephalosporin are the initial agents of choice [17]. Since the common organisms that tend to cause acute osteodiscitis are *Staphylococcus aureus*, *Staphylococcus epidermidis*, *Kingella kingae*, *Enterobacteriaceae*, and *Streptococcus pneumoniae*, an anti-*Staphylococcal *antibiotic and a third-generation cephalosporin are the initial agents of choice for their spectrum of activity. Duration of antibiotic therapy is variable and case-dependent [6].

We do not have official guidelines for the treatment of discitis in the case of children. Due to this, there is no clarity on the duration of treatment. There have been several recommendations in the literature. Intravenous therapy of duration anywhere from one to three weeks has been described followed by switching to oral antibiotics. Discontinuation of therapy is achieved upon resolution of symptoms and normalization of the child’s ESR and CRP, thus making duration of antibiotic therapy variable and case-dependent. In cases of cervical spine involvement, torticollis or dysphagia can occur. Treatment with antibiotics till clinical resolution of symptoms while trending clinical improvement and normalization of ESR and CRP has been effective in treating children in the absence of complications. Immobilization and analgesics provide symptomatic relief [18]. Since our patient had marked clinical improvement of pain and return of flexibility by the time of discharge, he did not require physical therapy. Biopsy tends to be reserved in uncertain cases or in those that have poor response to pain control measures and antibiotics. In cases of tuberculosis affecting the spine, quadriparesis has been noted [19]. The affected disc space may show bone fusion on repeat imaging after completion of treatment [7]. There is a lack of definitive guidelines for follow-up of children with discitis. In one study, it was noted that changes in the vertebral body tend to resolve by 24 months and recovery of disc is seen after 34 months [20].

## Conclusions

Discitis and osteodiscitis are rare in children. Due to the younger age group it tends to occur in, verbalization of symptoms by the children may not be accurate. The symptoms most often are subtle and non-alarming to the parent as well, thus making an early diagnosis challenging for the pediatrician. When the disease course tends to be subacute or chronic, bone loss and affliction of growth potential are commonly seen. Children tend to have a long duration of symptoms and disease progression by the time an accurate diagnosis is made. Our patient is being followed up clinically on a monthly basis till another repeat MRI is obtained to determine resolution per the radiologist's recommendation. This case brings to light the importance of an early workup of the child, which includes imaging as well in order to start timely treatment.
